# Accelerated Electron Transfer in Nanostructured Electrodes Improves the Sensitivity of Electrochemical Biosensors

**DOI:** 10.1002/advs.202102495

**Published:** 2021-10-19

**Authors:** Kaiyu Fu, Ji‐Won Seo, Vladimir Kesler, Nicolo Maganzini, Brandon D. Wilson, Michael Eisenstein, Boris Murmann, H. Tom Soh

**Affiliations:** ^1^ Department of Electrical Engineering Stanford University Stanford CA 94305 USA; ^2^ Department of Radiology Stanford University Stanford CA 94305 USA; ^3^ Department of Chemical Engineering Stanford University Stanford CA 94305 USA; ^4^ Chan Zuckerberg Biohub San Francisco CA 94158 USA

**Keywords:** aptamer, biosensors, electrochemical sensors, electron transfer, nanopores

## Abstract

Electrochemical biosensors hold the exciting potential to integrate molecular detection with signal processing and wireless communication in a miniaturized, low‐cost system. However, as electrochemical biosensors are miniaturized to the micrometer scale, their signal‐to‐noise ratio degrades and reduces their utility for molecular diagnostics. Studies have reported that nanostructured electrodes can improve electrochemical biosensor signals, but since the underlying mechanism remains poorly understood, it remains difficult to fully exploit this phenomenon to improve biosensor performance. In this work, electrochemical aptamer biosensors on nanoporous electrode are optimized to achieve improved sensitivity by tuning pore size, probe density, and electrochemical measurement parameters. Further, a novel mechanism in which electron transfer is physically accelerated within nanostructured electrodes due to reduced charge screening, resulting in enhanced sensitivity is proposed and experimentally validated. In concert with the increased surface areas achieved with this platform, this newly identified effect can yield an up to 24‐fold increase in signal level and nearly fourfold lower limit of detection relative to planar electrodes with the same footprint. Importantly, this strategy can be generalized to virtually any electrochemical aptamer sensor, enabling sensitive detection in applications where miniaturization is a necessity, and should likewise prove broadly applicable for improving electrochemical biosensor performance in general.

## Graphical abstract

In this work, nanostructured electrodes with tunable porosity for electrochemical biosensing that offer increased signal level and lower limit of detection than conventional planar electrodes are reported. Importantly, a novel mechanism in which electron transfer is physically accelerated within nanostructured electrodes due to reduced charge screening, resulting in enhanced sensitivity is proposed and experimentally validated.

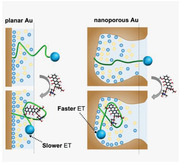

## 1 Introduction

Electrochemical biosensors have gained great interest in the past decade because they can be incorporated directly into very large‐scale integrated circuits.^[^ ^1^, ^2^, ^3^, ^4^ ^]^ This provides the exciting potential to fully couple biomolecular sensing with computation and communication in a miniaturized, low‐cost system.^[^ ^5^, ^6^, ^7^, ^8^, ^9^, ^10^, ^11^ ^]^ Sensitivity is a key consideration for many biomedical applications, because many clinical biomarkers are present at nanomolar to picomolar concentrations, and the biosensor must achieve sufficient sensitivity in a complex background of interferent molecules.^[^ ^12^, ^13^, ^14^, ^15^, ^16^, ^17^, ^18^, ^19^, ^20^ ^]^ Unfortunately, due to noise limitations in existing electronic measurement systems, the signal‐to‐noise ratio of conventional electrochemical biosensors degrades precipitously when they are miniaturized to the micron scale,^[^ ^21^ ^]^ reducing their sensitivity and making meaningful measurements of analyte concentrations challenging or even impossible in many cases.^[^ ^22^, ^23^ ^]^

There have been a number of advances in the fabrication of nanostructured electrodes over the last decade,^[^ ^24^, ^25^, ^26^, ^27^ ^]^ which achieve improved sensing properties relative to standard planar electrodes, such as increased signal levels and faster diffusion of redox species. In a seminal study, Kelley and co‐workers demonstrated that nanostructured electrodes with high surface curvature, which they termed “nanoflowers,” can greatly enhance DNA detection compared to planar electrodes, with limits of detection (LOD) in the femtomolar range.^[^ ^21^ ^]^ Seker and co‐workers have shown that similar improvements in sensitivity can also be achieved with nanoporous electrodes, with the additional benefit that the sensitivity and dissociation constant (*K*_D_) of the resulting sensors can be tuned by changing the size of the nanopores.^[^ ^28^, ^29^, ^30^ ^]^ While the majority of prior works with nanostructured electrodes have been limited to the hybridization‐based detection of nucleic acids, the use of electrode‐coupled aptamers as a molecular recognition element can extend the same detection strategy to small molecules, peptides, and proteins. Indeed, a few studies have demonstrated that the use of nanostructured electrodes can improve the sensitivity of aptamer‐based electrochemical sensors.^[^ ^22^, ^23^, ^31^, ^32^ ^]^ However, the mechanism behind this enhanced sensitivity remains unclear. Investigators have attributed the enhancement to simple geometric effects due to increased surface area from the nanostructures, but without a complete picture of the underlying mechanism, optimization of the design and manufacture of such aptamer‐based sensors remains challenging.

In this work, we identify a new mechanism underlying the improved sensitivity that can be achieved with nanoporous electrodes in electrochemical aptamer sensors, and exploit these findings in order to engineer electrodes that achieve greatly improved sensing performance. In our mechanistic model, these improvements in signal strength and sensitivity result from the altered charge screening within the nanoporous electrode structure, which arises due to changes in the “Debye volume” that defines the volume where the charge screening effect occurs. By decreasing this Debye volume within the confines of nanoscale porous structures, we can achieve more efficient electron transfer between the redox‐tagged aptamer, which mediates target recognition, and the surface of the gold electrode. We present computational models and experimental data that offer compelling evidence to support our hypothesis, and show that by tuning the electric double layer (EDL) —the region contained within the Debye volume—we can engineer the rate of electron transfer between our reporter and the electrode. Our experiments demonstrate that by tuning features such as the nanopore size, aptamer probe density, and electrochemical interrogation parameters, we can achieve an up to 24‐fold boost in signal and nearly fourfold improved LOD relative to an equivalent sensor employing planar electrodes. We subsequently propose and experimentally validate a mechanism underlying this improved signal output and sensitivity. In our model, these improvements result from weakened charge screening within the nanoporous electrode structure, enabling more efficient electron transfer between the redox‐tagged aptamer and the gold electrode. Based on this mechanism and our testing of different nanoporous electrode structures, we demonstrate the capability to tune the electrochemical sensors in terms of signal gain, LOD, or other performance metrics. The mechanistic principles identified in this work should be broadly applicable for improving the sensitivity of aptamer‐based electrochemical biosensors to a wide range of biomolecules in diagnostic and health monitoring applications.

## 2 Results and Discussion

### 2.1 Characterization of Aptamer‐Immobilized Nanoporous Electrodes

**Figure 1:**
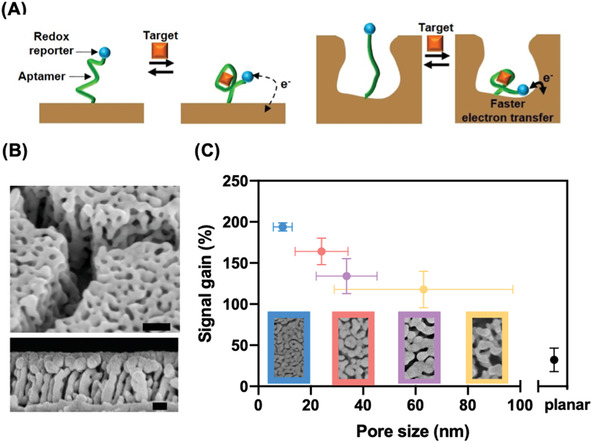
A) Schematics of electrochemical aptamer sensors on planar (left) and nanoporous (right) electrodes. The structure‐switching aptamer, end‐labeled with a methylene blue (MB) reporter, is unfolded in the absence of its target, doxorubicin (DOX). This situates MB far from the electrode, yielding minimal signal. DOX binding induces aptamer folding, bringing MB close to the electrode and producing an increase in current. B) Scanning electron microscopy (SEM) images of the nanoporous electrode. Upper and lower panels show top and side views, with scale bars of 100 and 50 nm, respectively. C) Impact of pore size on signal gain. Bottom panels show SEM images of the various nanoporous electrodes. Error bars in the *x*‐direction represent the standard deviation of different nanopores captured in the corresponding SEM images. Error bars in the *y*‐direction represent the standard deviation of the signal gain of three replicates.

As a proof‐of‐concept experiment for elucidating mechanisms of aptamer‐electrode interactions within nanostructured electrodes, we employed a sensor system in which we immobilized a well‐characterized doxorubicin (DOX) aptamer^[^ ^33^, ^34^, ^35^ ^]^ onto both planar and nanoporous gold electrodes via gold‐thiol interaction (**Figure** **1**A). In order to generate an electrochemical readout, the DOX aptamer was coupled to a methylene blue (MB) redox reporter. In the absence of target, the aptamer is generally in an unfolded state, limiting electron transfer between the reporter and the electrode. Target binding causes the aptamer to adopt a folded conformation, which brings the MB tag into closer proximity to the electrode surface and thus increases the electron transfer rate.

We measured the signal from each electrode using square‐wave voltammetry (SWV) as an indicator of conformational change of the aptamer upon binding DOX. SWV is widely used for electrochemical aptamer sensors, as it provides higher sensitivity and lower background than most other electrochemical techniques.^[^ ^36^, ^37^ ^]^ SWV works by applying a series of small voltage steps to create electric fields in the electrochemical cell. A single square voltage waveform is applied to create two phases at each step; the positive phase partially oxidizes the MB group coupled to the aptamer, and the negative phase reduces it. The current is measured near the end of each phase, and these currents are then subtracted from each other. Because the oxidative and reductive currents have opposite signs, this subtraction maximizes the faradaic current considered in the measurement. This difference between the two phases is greatest near MB's redox potential (*E* ^0^ = −0.21 V vs Ag/AgCl), creating a distinctive peak (Figure S1, Supporting Information). We characterize these peaks using two key metrics. The signal level reflects the absolute height of the SWV peak at a defined concentration, whereas the signal gain is the ratio between the signal level at a defined concentration and a target‐free control. Higher signal level helps distinguish SWV peaks from noise, while higher signal gain makes it easier to quantify different concentrations.

We fabricated the nanoporous electrodes using a dealloying process with Ag:Au alloys.^[^ ^38^, ^39^ ^]^ The pore size in these electrodes can be tuned by adjusting the ratio of Ag to Au. We used two approaches—thermal annealing^[^ ^28^ ^]^ and electrochemical coarsening^29^—to adjust the average pore and ligament sizes in our nanoporous structures. Using these two mechanisms, we were able to fabricate nanoporous electrodes with average pore sizes between 9.3 and 63.1 nm (Figure 1B). For comparison, we also fabricated planar electrodes with the same footprint (100 × 100 µm^2^). By controlling the nanopore size, we found that we could engineer the signal gain and improve the signal level of our sensor. We characterized the signal gain of nanoporous electrodes with different pore sizes after adding a saturating concentration (100 × 10^−6^ m) of DOX. Decreasing the pore size led to higher signal gain, where the smallest nanopores showed the highest signal gain of 194% versus 32% for the planar electrode (Figure 1C). We optimized for signal gain by utilizing the smallest average nanopore size (9.3 nm) for subsequent experiments.

Next, we optimized several of the control parameters for our nanoporous sensor. Changing the concentration of aptamer molecules applied during the immobilization process offers a way to control the probe density and inter‐aptamer spacing on the electrode surface.^[^ ^40^ ^]^ High aptamer density leads to steric hindrance, which impedes target binding, whereas excessively low aptamer density can cause the signal level to decrease to undetectable levels. We prepared nanoporous and planar electrodes using five different aptamer concentrations (0.1, 0.5, 1, 3, 10× 10^−6^ m), yielding molecular probe densities that we labeled as d0.1, d0.5, d1, d3, and d10, respectively (refer to Figure S2 for details, Supporting Information). Then we generated calibration curves by measuring SWV at several concentrations of DOX, ranging from 100 × 10^−9^ m to 100 × 10^−6^ m. In parallel, we also optimized the frequency and amplitude of the waveform applied to the electrochemical cell during measurement, parameters that affect transduction from the redox reporter to the electrode.^[^ ^41^ ^]^ For each aptamer concentration, we performed SWV with five different frequencies (50, 100, 200, 300, and 400 Hz) and three different amplitudes (20, 50, and 100 mV) (refer to Figure S3 for details, Supporting Information).

**Figure 2:**
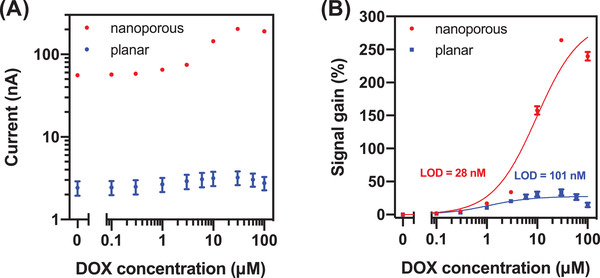
A) Signal level and B) signal gain of electrochemical aptamer sensors employing nanoporous (red) and planar (blue) electrodes in the presence of 100 × 10^−9^ m–100 × 10^−6^ m DOX. Plots are averaged over three replicates. Error bars represent the standard deviation.

The nanoporous electrodes consistently produced greatly improved performance relative to planar electrodes. We optimized both the nanoporous and planar sensor for the abovementioned conditions across several metrics: signal gain, signal level, and LOD (**Table** **1**). At 10 × 10^−6^ m DOX—the upper limit of the clinically‐relevant range for this drug^34^—we could independently improve signal gain by as much as three‐fold (from 59% to 179%) and signal level by as much as 24‐fold (from 30.4 to 728.0 nA) for the nanoporous electrodes relative to their planar counterparts. This superior performance can be partly explained by the much larger surface area achieved with the nanoporous electrode while keeping the same footprint as a planar electrode. This allows more total probes to be immobilized, which generates higher currents. At the same time, having more probes on the electrode includes more binding events in the ensemble measurement, lowering the variance of the measured signal. This is reflected in the smaller error between nanoporous replicates (≈4% CV) versus planar replicates (≈20% CV) (**Figure** **2**A; refer to Table S2 for details, Supporting Information). In combination with the increased signal gain that we observed for our electrodes, the lower variation and increased signal level result in a decreased LOD (Figure 2B). Indeed, by leveraging the additional signal gain and signal level, the sensor LOD could be decreased nearly four‐fold, from 101.3 ± 16.8 × 10^−9^ m on planar electrodes to 28.5 ± 1.4 × 10^−9^ m on nanoporous electrodes.

**Table 1:** Conditions yielding maximum sensor signal gain, signal level, and LOD for nanoporous and planar electrodes. Signal gain and level measurements are for 10 × 10^−6^ m DOX; enhancement ratio describes improved performance in each optimized metric for nanoporous versus planar sensors

Optimized metrics	Electrode	Probe density	SWV Hz	SWV Amp	Signal gain	Signal level [nA]	LOD [× 10^−6^ m]	Enhancement ratio
Signal gain	Nanoporous	d1	400 hz	20 mV	179.00%	180.9	0.080	3.0
	Planar	d1	200 hz	20 mV	59.37%	2.7224	0.273	
Signal level	Nanoporous	d10	400 hz	100 mV	76.14%	727.99	0.116	23.9
	Planar	d10	400 hz	100 mV	15.26%	30.415	0.107	
LOD	Nanoporous	d1	300 hz	20 mV	157.69%	143.72	0.028	3.6
	Planar	d3	100 hz	50 mV	30.78%	3.1551	0.100	

### 2.2 Mechanistic Explanation of Signal Enhancement

A number of researchers have reported increases in signal gain with nanostructured electrodes^[^ ^22^, ^23^, ^31^ ^]^ but without a definitive mechanistic explanation for this phenomenon. Some studies have suggested that the increased sensitivity is due to the change in the “effective *K*_D_” of the molecular probe, resulting from local increases in analyte concentration.^[^ ^21^, ^28^ ^]^ Although this maybe valid under certain conditions,^[^ ^42^ ^]^ our data from nanoporous and planar electrodes showed increased signal gain without a significant change in binding thermodynamics (*K*_D_ = 1.71 and 2.14 × 10^−9^ m, respectively).

**Figure 3:**
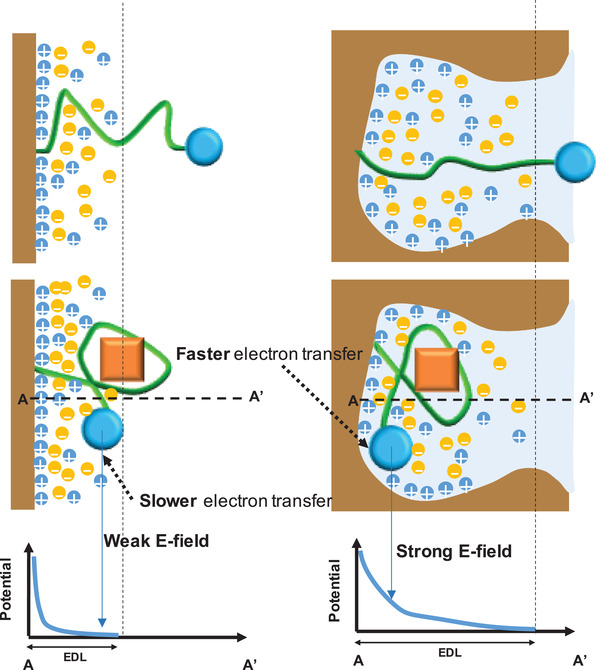
Scheme of how the electric double layer (EDL) from a planar (left) and nanoporous electrode (right) affects electron transfer from a MB reporter (blue circle) tethered to the aptamer (green) before and after target (orange square) binding. During electrochemical measurement, the MB reporter interacts with the EDL (shaded blue region), where a closer distance between the reporter and the electrode surface leads to faster electron transfer. In the nanoporous electrode, the MB group experiences stronger electric fields. A and A’ represent the electrode surface and the maximum distance of MB from the electrode, respectively.

As an alternative, we hypothesized that the nanoporous structure of our electrodes was directly affecting the kinetics of electron transfer between the MB reporter and the electrode through its weakening effect on charge screening. In electrochemical aptamer sensors that use SWV, electric fields are applied across the electrochemical cell to initiate concentration‐dependent electron transfer. However, in physiological samples and other electrolytic solutions, these electric fields are confined to the EDL, a small region adjacent to the electrode where ions screen the field being applied. This EDL is quite small—on the order of the Debye length (<1 nm)—and defines a small volume that the MB reporter must enter for electron transfer to occur. This can be approximated in terms of the Debye volume: the space between the electrode surface and an imaginary surface one Debye length normal to it. It has been shown that limiting the Debye volume at an electrode surface extends the EDL farther into the solution, thereby lessening the extent of electric field screening at that interface.^[^ ^43^, ^44^ ^]^ Consequently, the stronger electric fields within the nanopores increase the probability of a faradaic electron transfer event for a given conformation of an aptamer probe. Indeed, because of their high density of nanoscale features, nanoporous electrodes offer exactly the type of interface where screening is weaker and where we would predict faradaic electron transfer to be accelerated (**Figure** **3**).

**Figure 4:**
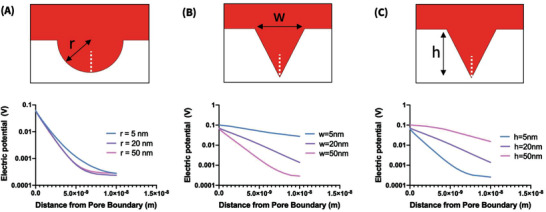
Simulations of screening for nanostructured electrode geometries with 100 mV applied. Top panels show the tested geometries, including A) semicircular pores of varying radius, and triangular pores of B) fixed height (20 nm) and varying width (w) or (C) fixed width (20 nm) and varying height (h). Bottom panels show electric potentials for a 10 nm cut line extending vertically from the deepest point in the structure (white dashed line in top panels).

We carried out a 2D simulation study in COMSOL to test this hypothesis. Because the morphology of nanoporous electrodes is irregular and highly variable, we simplified our study by simulating several basic geometries that we believe are representative of geometric elements typically found in true nanoporous electrode structures. We focused on semicircles with radii of 5, 20, and 50 nm (**Figure** **4**A) and triangles with base widths and heights of 5, 20, and 50 nm (Figure 4B,C). We applied 100 mV to the interfaces under study, approximating the screening conditions in the cell at the redox potential of MB. For each structure, the electric potential was extracted for a 10 nm straight line from the structure's apex to evaluate potential decay in space. Typically, any applied potential will decay exponentially with distance, with a spatial rate constant defined by the Debye length. However, our simulation suggested that nanoporous gold surfaces could diminish the effects of screening, depending on the size of the cavities involved. As critical dimensions decreased in size, the potential decayed less sharply with distance, indicating that the EDL is extended in the nanostructure and that screening is weakened. Semicircles exhibited stronger screening than triangular shapes, and this is likely due to the sides of the triangles creating closer distances between adjacent surfaces, mimicking nanogap structures.^[^ ^45^ ^]^ Indeed, our simulation results provide strong evidence that the spatial scale of the nanopore structures in our electrodes is sufficient to support weakened charge screening relative to a planar surface.

**Figure 5:**
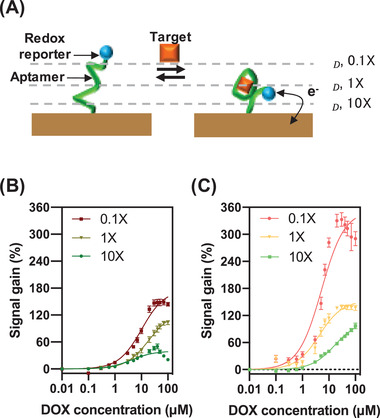
A) Ionic strength affects the EDL configuration, which affects transduction between the MB reporter and the electrode. As the ionic strength of the solution increases, the Debye length (*λ*_D_) decreases. Calibration curves were generated by performing SWV on aptamer‐functionalized B) planar and C) nanoporous electrodes with samples spiked with varying concentrations of DOX in different dilutions of SSC buffer to assess the impact of ionic strength on signal gain. Datapoints are averaged over three replicates, and error bars show standard deviation.

To experimentally test the effect of weakened screening on electrochemical measurements, we varied the ionic strength of the sample and generated calibration curves by performing SWV on the aptamer‐functionalized nanoporous electrodes. Ionic strength is known to act as a “control knob” for screening at electrode‐electrolyte interfaces, where the relationship between the ionic strength (*I*) and Debye length (*λ*_D_) is $\lambda_{D}\propto \frac{1}{\sqrt{I}}$. Thus, by tuning the ionic strength of the sample, we can modulate the screening conditions at the electrode interface to study how screening affects electron transfer from MB to the electrode (**Figure** **5**A). We prepared samples containing various concentrations of DOX in 0.1X, 1X, or 10X saline‐sodium citrate (SSC) buffer, where 1X SSC buffer contains 150 × 10^−3^ m NaCl and 15 × 10^−3^ m trisodium citrate (pH 7.0). We then measured square‐wave voltammograms with planar (Figure 5B) and nanoporous electrodes (Figure 5C) in each sample to generate a calibration curve for each buffer concentration (refer to Figure S4 for details of the effect of ionic strength on measurement, Supporting Information). We note that at high analyte concentrations, we observed a decreasing signal gain under some experimental conditions. A similar trend was also observed in a recent study, and this is likely due to the interference of the target with electron transfer between the target‐aptamer complex and the electrode surface.^[^ ^31^ ^]^

These calibration curves confirmed our theory—as the ionic strength of the solution decreases, the EDL extends farther into the solution and signal gain increases for both planar and nanoporous electrodes. This is consistent with our simulations, which showed that the EDL extends farther in smaller geometries (Figure 4), and our data showing that nanoporous electrodes with smaller pores generate higher signal gain (Figure 1C). Notably, the effective *K*_D_ of the sensor was also affected by changes in ionic strength. This effect is likely a consequence of charge screening as well, because electrostatic changes alter the intramolecular (e.g., energetics of Watson–Crick base pairing, formation of secondary and tertiary structures) and intermolecular interactions (e.g., binding energy) that determine the thermodynamics of the aptamer sensor.

**Figure 6:**
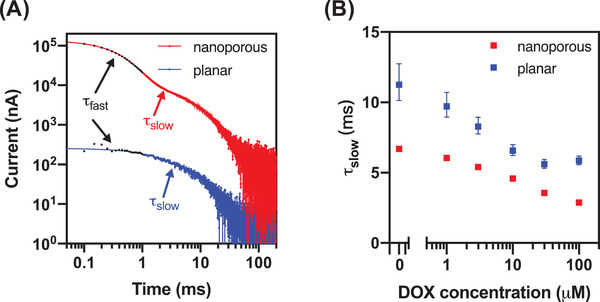
A) Chronoamperograms of planar (blue) and nanoporous (red) electrode sensors with an electrode size of 500 × 500 µm^2^ in the absence of DOX (log scale, negative values not shown). B) The decay time (*τ*_slow_) of both electrodes at various DOX concentrations. Error bars represent the confidence intervals of the curve fitting.

Finally, we further validated our hypothesis by generating chronoamperograms to show that weakened screening in the nanoporous electrodes indeed causes accelerated electron transfer. We tested aptamer‐functionalized nanoporous electrodes with several concentrations of DOX up to 100 × 10^−3^ m, averaging 50 measurements from each electrode to reduce noise at the lower current ranges. These chronoamperogram measurements are made up of two types of current: non‐faradaic current, which captures the movement of ions to charge the EDL, and faradaic current, which captures the electrochemical transfer of charge to or from redox reporters. The total current can be modeled as a two‐phase exponential decay, where the faster phase (*τ*_fast_) represents the capacitive EDL charging current and the slower phase (*τ*_slow_) represents the electrochemical current (**Figure** **6**A).^[^ ^46^ ^]^ With increasing concentrations of DOX, we expect more aptamers to be bound to their target, and we predictably observed that *τ*_slow_ became shorter with increasing target concentrations. This indicates that more charge is transferred sooner as the aptamer binds to its target. We found that *τ*_slow_ was consistently shorter across DOX concentrations for nanoporous electrodes compared to planar electrodes, indicating faster faradaic reactions between the redox reporter and electrode (Figure 6B).

This observation is consistent with the core mechanism of structure‐switching electrochemical aptamer sensors, in which charges are exchanged when the redox reporter encounters sufficiently high electric fields within the electrode's EDL. If a binding event brings the MB reporter closer to the surface (on average), the faradaic reaction will complete faster. Importantly, our data demonstrates that the extended EDL on the nanoporous electrodes also facilitates more efficient electron transfer than on the planar electrodes.

## 3 Conclusion

In this work, we have developed and experimentally validated a model to explain why nanoporous gold electrodes can deliver considerably improved performance relative to planar gold surfaces in the context of electrochemical aptamer sensors. We first showed that nanoporous electrodes consistently offer superior signal output and signal gain relative to planar surfaces in an electrochemical aptamer sensor for the chemotherapeutic drug doxorubicin, and show that these electrodes can be optimized to achieve ≈24‐fold higher signal level and approximately fourfold lower LOD relative to planar gold electrodes. We subsequently hypothesized that this greater sensitivity and signal output may be the consequence of the decreased Debye volume and reduced impact of charge screening within these nanopores, and were able to test and confirm this hypothesis via both computer simulations and experimental testing. Collectively, our results reveal design principles that can guide the production of electrochemical aptamer sensors with optimized detection performance—for example, employing smaller nanopores to maximize the signal gain generated in response to target binding, or tuning the square‐wave voltammetry settings to achieve the best LOD. We believe that this ability to engineer electron transfer efficiency should prove highly valuable for improving the performance of electrochemical biosensors for a wide range of biomolecules in diagnostic and health monitoring applications.

## 4 Experimental Section

### Reagents and Materials

The DOX aptamer was adapted from the previous studies^[^ ^34^, ^35^ ^]^ and obtained from Biosearch Technologies: 5’‐HS‐C6‐ACCATCTGTGTAAGGGGTAAGGGGTGGT‐MB‐3’, where MB indicates the methylene blue reporter. 6‐mercapto‐1‐hexanol (6‐MCH), tris(2‐carboxyethyl)phosphine (TCEP), and DOX were obtained from Sigma‐Aldrich. A 1X stock solution of SSC buffer was prepared by dilution of SSC stock solution (20X, pH 7.4, Thermo Fisher Scientific) with nuclease‐free water. DOX solution was prepared before measurement in SSC buffer.

### Device Fabrication and Characterization

A 100 × 100 µm^2^ Ti/Au/Ag:Au (10/50/300 nm) film was patterned onto glass slides via lift‐off process. The ratio of cosputtered Ag:Au film was 2:1.^[^ ^28^, ^29^ ^]^ Next, Ag was selectively etched by nitric acid (70% v/v) for 5 min, forming the Au nanoporous microelectrode. Finally, the whole area except the 100 × 100 µm^2^ Au nanoporous microelectrode was encapsulated (see Figures S5 and S6 for characterization details, Supporting Information).

Post‐treatment of the fabricated nanoporous electrode slide included either a thermal annealing process or an electrochemical coarsening process (Table S1, Supporting Information). The former process entails treating the nanoporous electrode slide at 230 °C for 10 min on a hotplate. This resulted in larger nanopores and fewer cracks; the average pore size was 9.29 nm at room temperature (RT), but increased to 24.12 nm at 230^ ^°C. Electrochemical coarsening is achieved through cyclic voltammetry (CV) in 0.5 m sulfuric acid solution with a potential window of 0.3–1.2 V for multiple scans. The resulting nanostructures were characterized by scanning electron microscopy (FEI Nova NanoSEM 450). The surface of the nanoporous gold was repetitively oxidized and reduced to form larger pore sizes ranging from 9.29 to 33.7 nm. By performing both thermal annealing and electrochemical coarsening, the pore size up to 63.11 nm could be increased. CV of nanoporous and planar electrodes was conducted in 0.05 m sulfuric acid at a scan rate of 50 mV s^−1^ over the potential range −0.25 to +1.75 mV to determine the effective surface area of the electrode (Figure S7, Supporting Information).^[^ ^28^ ^]^

### Electrochemical Aptamer Sensor Characterization

After rinsing the nanoporous electrode slide with deionized (DI) water, the freshly prepared slide was incubated with 1 × 10^−6^ m aptamer in 1X SSC buffer for one hour. Soft‐polymer PDMS wells were sealed on top of the nanoporous electrode slide to hold each electrode's solution. To study the effects of aptamer surface density, other concentrations of DOX aptamer were also prepared. Before incubation, 100 × 10^−6^ m aptamer in DI water was reacted with a 1000‐fold excess of TCEP solution to produce free thiol groups for aptamer immobilization. After immobilization, the nanoporous electrode slide was washed with excess buffer and then incubated with 10 × 10^−3^ m 6‐MCH for 2 h to passivate the remaining electrode surface. The electrode was then stored in 1X SSC before electrochemical measurement.

All measurements were performed in PDMS wells with a PalmSens 4 potentiostat. All working electrodes—whether nanoporous or planar—were 100 × 100 µm^2^ unless specified otherwise. Commercial Ag/AgCl reference electrodes and Pt wire counter electrodes were from CH Instruments. SWV was carried out in 1 × SSC buffer over the potential range of −0.5 to 0.0 V with an amplitude of 20–100 mV, step‐size of 2 mV, and pulse frequencies ranging from 50 to 400 Hz. DOX binding curves were generated across various probe densities and SWV parameters as part of the optimization. We also calculated the LOD for DOX based on the concentration that gave a signal three baseline standard deviations (*σ*_b_) above zero

(1) $$\mathrm{LOD}=K_{D}*3\sigma_{b}/\left(B_{\max}-3\sigma_{b}\right)$$

where *K*_D_ and *B*_max_ are the dissociation constant and maximum specific binding, respectively. These values were obtained by fitting the signal gain at different DOX concentrations to a Langmuir isotherm.^[^ ^47^ ^]^

### COMSOL Simulation Methodology

Simulations were done in COMSOL 5.5 using the Electrochemistry module. By coupling the “electrostatics” and “transport of dilute species” physics, the Poisson–Nernst–Planck system of equations was solved for a 100 × 10^−3^ m NaCl electrolyte with Debye length ≈1 nm. Boundary conditions were no flux at the interface under study, to define the electrode as polarizable and eliminate effects of any faradaic reactions, and equilibrium concentrations were defined to mimic a controlled potential state as established by a potentiostat or similar circuitry. Shapes were simulated as part of a large square field with a side length of 1 µm, to prevent any effects due to other boundaries.

## Conflict of Interest

The authors declare no conflict of interest.

## Author Contributions

K.F., J.‐W.S., and V.K. contributed equally to this work. K.F., J.W.S., and H.T.S. initiated the project. K.F. and J.W.S. designed and characterized the device. K.F., J.W.S., and V.K. designed the experiments. K.F. executed the experiments and collected the data. V.K. conceived the theoretical model and performed the simulations. K.F. and V.K. analyzed the data. K.F., V.K., J.W.S., and H.T.S. wrote the paper. All authors discussed the data and edited the paper.

## Supporting information

Supporting Information

## Data Availability

The data that support the findings of this study are available from the corresponding author upon reasonable request.
