# Supplementary material for: Accelerated Electron Transfer in Nanostructured Electrodes Improves the Sensitivity of Electrochemical Biosensors
Source: Adv Sci (Weinh). 2021 Oct 19;8(23):2102495. doi: 10.1002/advs.202102495 (PMC8655170; doi:10.1002/advs.202102495)
Supplement: Supplementary file 1 — Supporting Information [file ADVS-8-2102495-s001.pdf]

© 2021 Wiley-VCH GmbH

ADVANCED SCIENCE
Open Access

Supporting Information

for *Adv. Sci.,* DOI: 10.1002/advs.202102495

Accelerated electron transfer in nanostructured electrodes improves the sensitivity of electrochemical biosensors

*Kaiyu Fu, Ji-Won Seo, Vladimir Kesler, Nicolo Maganzini, Brandon D. Wilson, Michael Eisenstein, Boris Murmann, and H. Tom Soh**

Supplementary Information

Accelerated electron transfer in nanostructured electrodes improves the sensitivity of electrochemical biosensors

K. Fu et al.

Contents

Supplementary **Table S1 |** Summary of properties for planar and nanoporous gold electrodes produced under different conditions.

Supplementary **Table S2 |** Coefficients of variation (CV) of signal level for planar and nanoporous gold electrodes obtained from different target concentrations.

Supplementary **Figure S1 |** Representative SWV plots from nanoporous (left) and planar electrodes (right) comparing buffer-only signal with signal from 10 μM DOX.

Supplementary **Figure S2 |** Aptamer probe density on nanoporous and planar electrodes after varying the incubated aptamer concentration from 100 nM to 10 μM.

Supplementary **Figure S3 |** Optimization of SWV parameters.

Supplementary **Figure S4 |** Signal gain at different ionic strengths.

Supplementary **Figure S5 |** Representative SEM images and EDS analysis from nanoporous and planar electrode.

Supplementary **Figure S6 |** Representative AFM images from nanoporous and planar electrode.

Supplementary **Figure S7 |** Representative cyclic voltammograms from nanoporous (red) and planar electrodes (blue), where the reduction peaks indicate the gold electrode surface area.

Supplementary **Table S3 |** Comparison of existing doxorubicin biosensors for various detection methods.

	Average pore size [nm]	Signal gain at 100 µM DOX [%]
**Planar electrode**	**Non-porous**	**32.3 ± 14.5**
**No post-treatment**	**9.3 ± 3.6**	**193.8 ± 5.1**
**Thermal annealing only**	**24.1 ± 10.1**	**159.7 ± 16.1**
**Electrochemical coarsening only**	**33.7 ± 11.6**	**155.0 ± 21.3**
**Thermal annealing with electrochemical coarsening**	**63.1 ± 34.1**	**145.0 ± 22.2**

Table S1: Summary of properties for planar and nanoporous gold electrodes produced under different conditions.

Target concentration [μM]	Nanoporous CV[%]	Planar CV[%]
**0.1**	**3.85**	**19.59**
**0.3**	**3.87**	**19.56**
**1.0**	**3.47**	**19.72**
**3.0**	**3.37**	**19.82**
**10.0**	**2.47**	**19.53**
**30.0**	**3.84**	**18.84**
**100.0**	**5.21**	**18.30**

Table S2: Coefficients of variation (CV) of signal level for planar and nanoporous gold electrodes obtained from different target concentrations. Data are averaged from six replicates (n = 6).

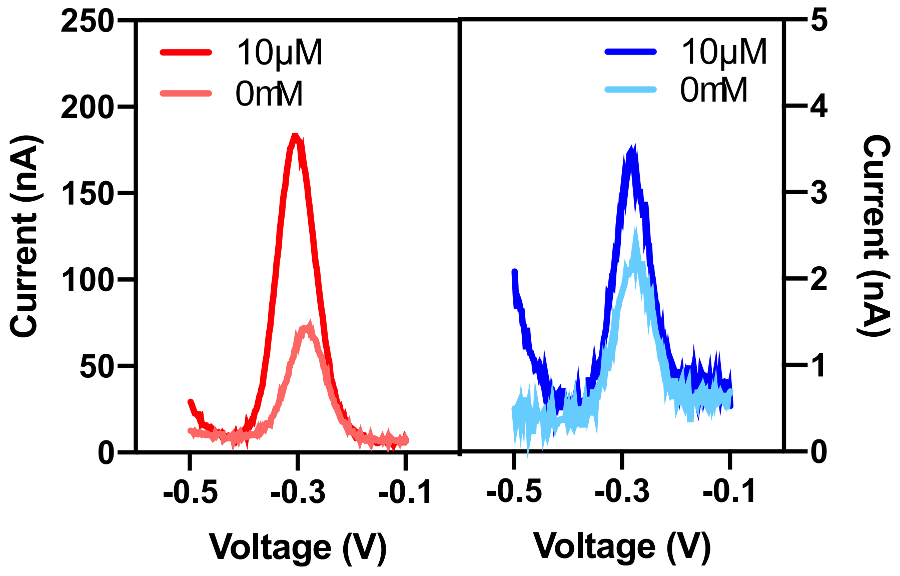

Figure S1. Representative SWV plots from nanoporous (left) and planar electrodes (right) comparing buffer-only signal with signal from 10 µM DOX.

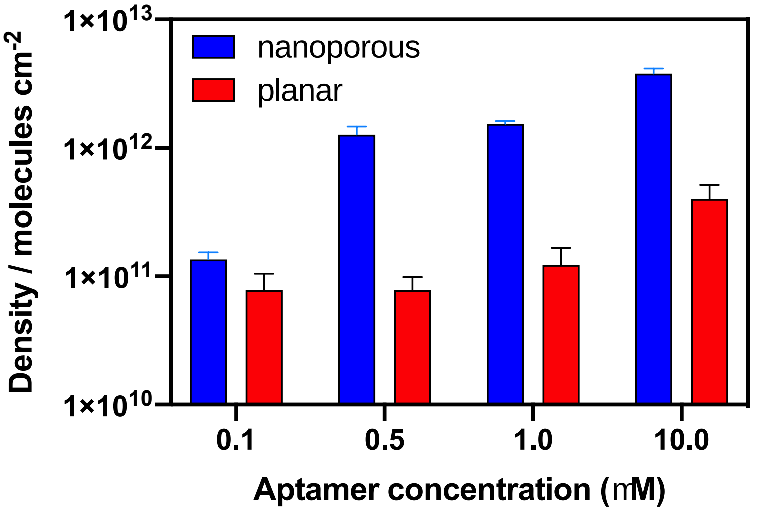

Figure S2. Aptamer probe density on nanoporous and planar electrodes after varying the incubated aptamer concentration from 100 nM to 10 μM. Datapoints are averages from seven replicates (n = 7).

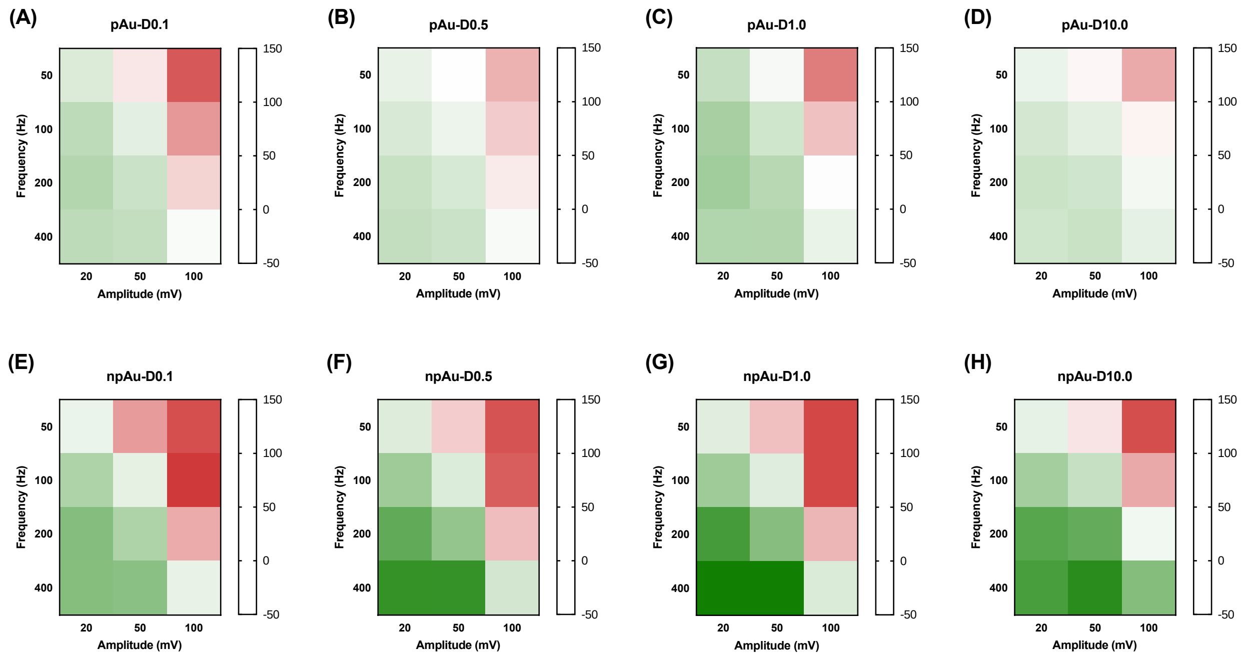

Figure S3. Optimization of SWV parameters. Heat-map of signal gain of electrochemical sensors with nanoporous (**A** to **D**) or planar electrodes (**E** to **H**) at different frequencies (50–400 Hz) and amplitudes (20–100 mV). Panels from left to right represent a shift from low to high aptamer density. All data are averaged over six replicates (n = 6).

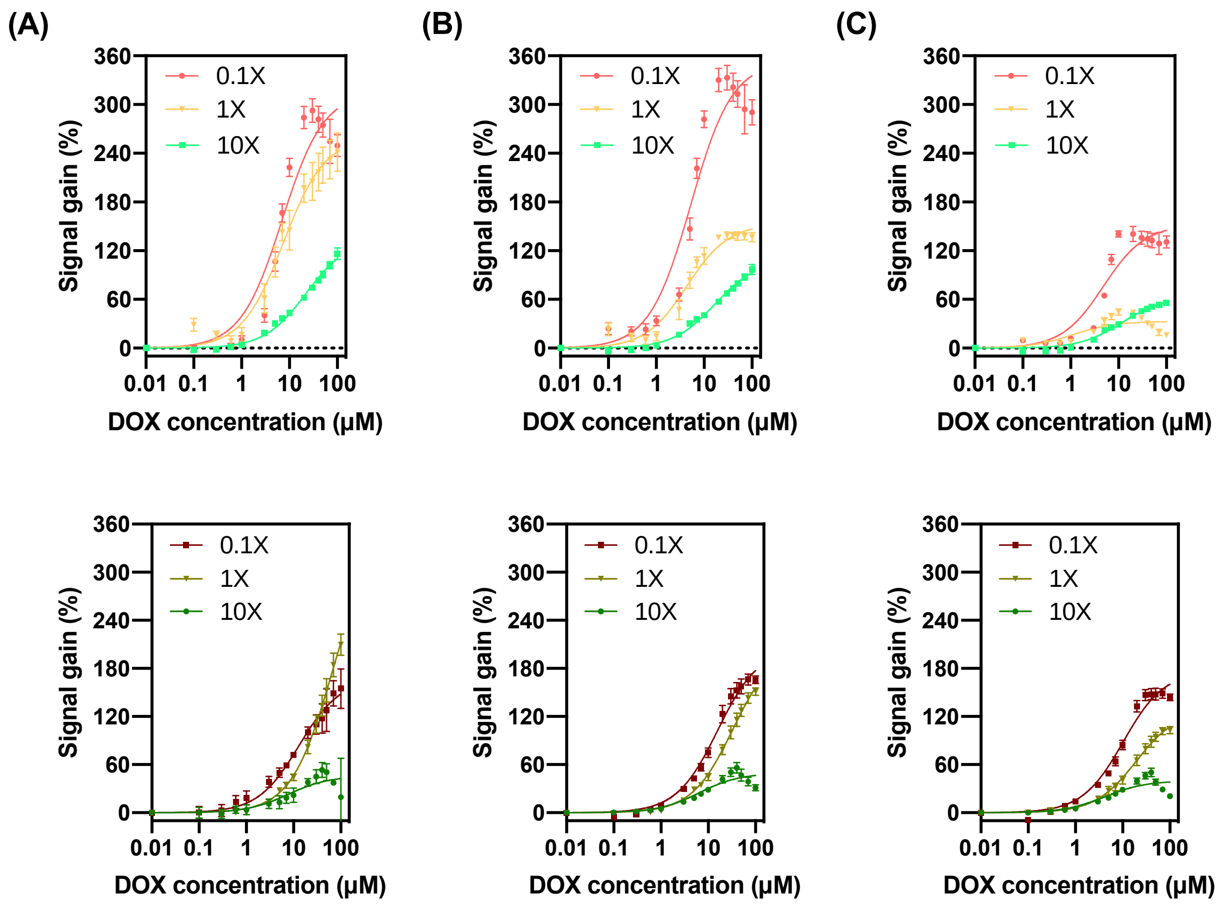

Figure S4. Signal gain at different ionic strengths. Signal gain is shown from nanoporous (top) and planar (bottom) electrodes in 0.1X, 1X and 10X SSC buffer at three SWV frequencies: (**A**) 400 Hz, (**B**) 200 Hz, and (**C**) 100 Hz. Datapoints are averaged over three replicates (n = 3). Notably, the effects of changing ionic strength on SWV were visible at lower frequencies on planar electrodes than on nanoporous electrodes; at higher frequencies, the signal gain curves of both types of electrodes converged towards their maximum values regardless of ionic strength. We speculate that these differences are caused by the difference in morphology between the flat planar and curved nanoporous interfaces.

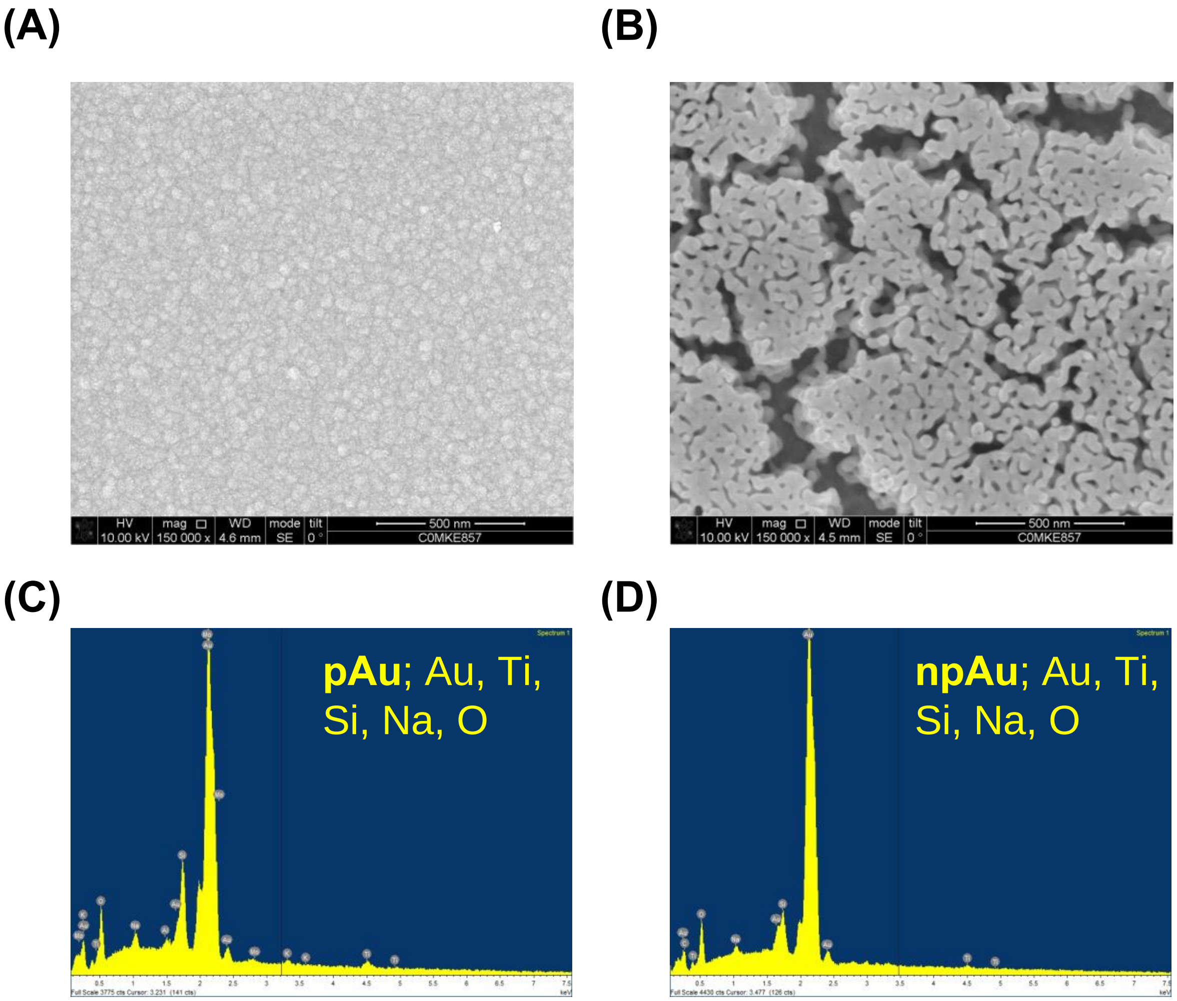

Figure S5. Representative (**A, B**) scanning electron microscopy (SEM) images and (**C, D**) energy dispersive X-ray spectroscopy (EDS) analysis of planar (left) and nanoporous (right) electrodes. EDS analysis confirmed that the nanoporous electrode is made of gold and free of silver residue after the wet etching process. Si, Na, O traces are the from the substrate glass supporting the electrode, and Ti is the adhesion layer between the substrate glass and gold electrode; these elemental signals are roughly equivalent for both electrodes.

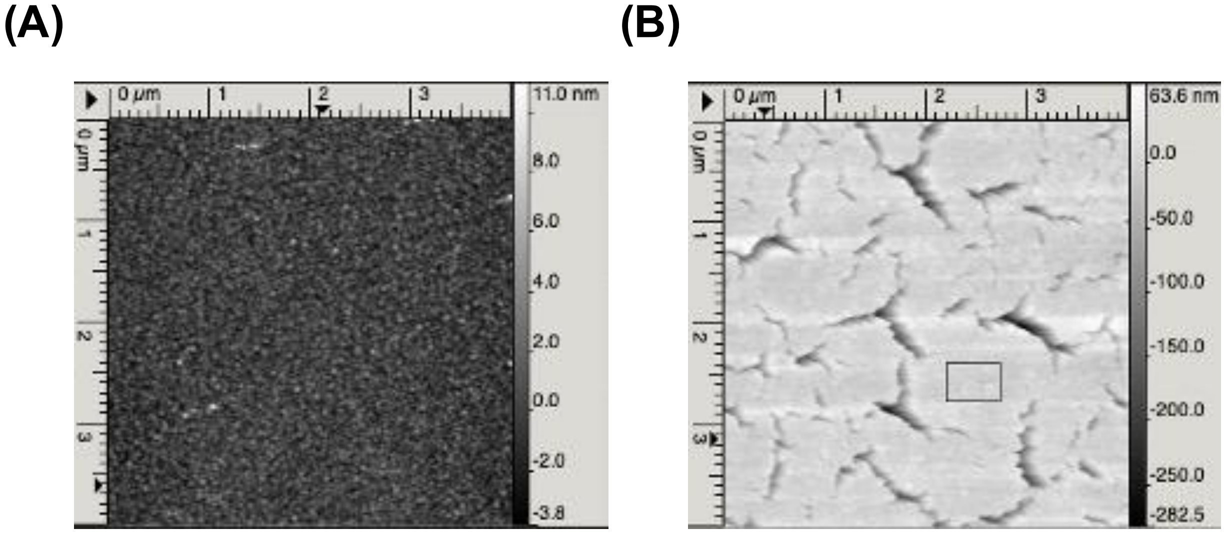

Figure S6. Representative AFM images from (A) planar and (B) nanoporous electrodes. AFM topography measurements were taken in AC (tapping) mode. Root mean square surface roughness (R_q) was considerably higher for the nanoporous electrodes (R_q = 29.23 nm) than the planar electrodes (R_q = 1.12 nm), even when we analyzed a region of the nanoporous surface away from the larger porous features (black rectangle; R_q = 7.22 nm).

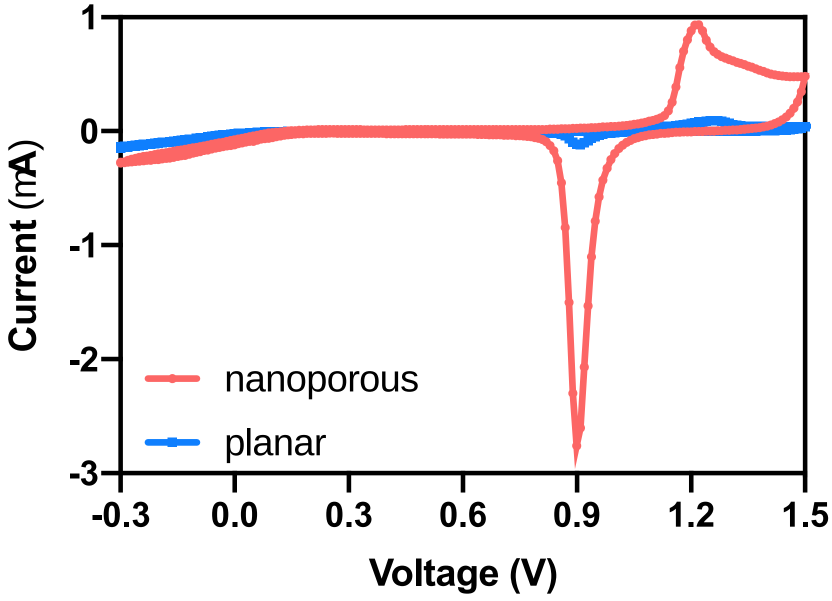

Figure S7. Representative cyclic voltammograms from nanoporous (red) and planar electrodes (blue), where the reduction peaks indicate the gold electrode surface area.

REFERENCES

1. Benyettou, F.; Alhashimi, M.; O'Connor, M.; Pasricha, R.; Brandel, J.; Traboulsi, H.; Mazher, J.; Olsen, J.-C.; Trabolsi, A., Sequential Delivery of Doxorubicin and Zoledronic Acid to Breast Cancer Cells by CB[7]-Modified Iron Oxide Nanoparticles. *ACS Applied Materials & Interfaces* **2017,** *9* (46), 40006-40016.
2. Zhang, S.; Huang, C.; Li, Z.; Yang, Y.; Bao, T.; Chen, H.; Zou, Y.; Song, L., Comparison of pharmacokinetics and drug release in tissues after transarterial chemoembolization with doxorubicin using diverse lipiodol emulsions and CalliSpheres Beads in rabbit livers. *Drug Delivery* **2017,** *24* (1), 1011-1017.
3. Roszkowska, A.; Tascon, M.; Bojko, B.; Goryński, K.; dos Santos, P. R.; Cypel, M.; Pawliszyn, J., Equilibrium ex vivo calibration of homogenized tissue for in vivo SPME quantitation of doxorubicin in lung tissue. *Talanta* **2018,** *183*, 304-310.
4. Scheeren, L. E.; Nogueira-Librelotto, D. R.; Fernandes, J. R.; Macedo, L. B.; Marcolino, A. I. P.; Vinardell, M. P.; Rolim, C. M. B., Comparative Study of Reversed-Phase High-Performance Liquid Chromatography and Ultraviolet–Visible Spectrophotometry to Determine Doxorubicin in pH-Sensitive Nanoparticles. *Analytical Letters* **2018,** *51* (10), 1445-1463.
5. Jouyban, A.; Samadi, A.; Jouyban-Gharamaleki, V.; Khoubnasabjafari, M., A microscale spectrophotometric method for quantification of doxorubicin in exhaled breath condensate. *Analytical Methods* **2019,** *11* (5), 648-653.
6. Krüger, H. R.; Schütz, I.; Justies, A.; Licha, K.; Welker, P.; Haucke, V.; Calderón, M., Imaging of doxorubicin release from theranostic macromolecular prodrugs via fluorescence resonance energy transfer. *Journal of Controlled Release* **2014,** *194*, 189-196.
7. Fan, Z.; Sun, L.; Huang, Y.; Wang, Y.; Zhang, M., Bioinspired fluorescent dipeptide nanoparticles for targeted cancer cell imaging and real-time monitoring of drug release. *Nature Nanotechnology* **2016,** *11* (4), 388-394.
8. Sparks, H.; Kondo, H.; Hooper, S.; Munro, I.; Kennedy, G.; Dunsby, C.; French, P.; Sahai, E., Heterogeneity in tumor chromatin-doxorubicin binding revealed by in vivo fluorescence lifetime imaging confocal endomicroscopy. *Nature Communications* **2018,** *9* (1), 2662.
9. Galievsky, V.; Pawliszyn, J., Fluorometer for Screening of Doxorubicin in Perfusate Solution and Tissue with Solid-Phase Microextraction Chemical Biopsy Sampling. *Analytical Chemistry* **2020,** *92* (19), 13025-13033.
10. Farhane, Z.; Bonnier, F.; Casey, A.; Byrne, H. J., Raman micro spectroscopy for in vitro drug screening: subcellular localisation and interactions of doxorubicin. *Analyst* **2015,** *140* (12), 4212-4223.
11. Panikar, S. S.; Banu, N.; Escobar, E.-R.; García, G.-R.; Cervantes-Martínez, J.; Villegas, T.-C.; Salas, P.; De la Rosa, E., Stealth modified bottom up SERS substrates for label-free therapeutic drug monitoring of doxorubicin in blood serum. *Talanta* **2020,** *218*, 121138.
12. Kalambate, P. K.; Li, Y.; Shen, Y.; Huang, Y., Mesoporous Pd@Pt core–shell nanoparticles supported on multi-walled carbon nanotubes as a sensing platform: application in simultaneous electrochemical detection of anticancer drugs doxorubicin and dasatinib. *Analytical Methods* **2019,** *11* (4), 443-453.
13. Yan, F.; Chen, J.; Jin, Q.; Zhou, H.; Sailjoi, A.; Liu, J.; Tang, W., Fast one-step fabrication of a vertically-ordered mesoporous silica-nanochannel film on graphene for direct

and sensitive detection of doxorubicin in human whole blood. *Journal of Materials Chemistry C* **2020,** *8* (21), 7113-7119.
14. Sharifi, J.; Fayazfar, H., Highly sensitive determination of doxorubicin hydrochloride antitumor agent via a carbon nanotube/gold nanoparticle based nanocomposite biosensor. *Bioelectrochemistry* **2021,** *139*, 107741.
15. Ferguson, B. S.; Hoggarth, D. A.; Maliniak, D.; Ploense, K.; White, R. J.; Woodward, N.; Hsieh, K.; Bonham, A. J.; Eisenstein, M.; Kippin, T. E.; Plaxco, K. W.; Soh, H. T., Real-Time, Aptamer-Based Tracking of Circulating Therapeutic Agents in Living Animals. *Science Translational Medicine* **2013,** *5* (213), 213ra165.
16. Li, H.; Arroyo-Currás, N.; Kang, D.; Ricci, F.; Plaxco, K. W., Dual-Reporter Drift Correction To Enhance the Performance of Electrochemical Aptamer-Based Sensors in Whole Blood. *Journal of the American Chemical Society* **2016,** *138* (49), 15809-15812.
17. Li, H.; Dauphin-Ducharme, P.; Arroyo-Currás, N.; Tran, C. H.; Vieira, P. A.; Li, S.; Shin, C.; Somerson, J.; Kippin, T. E.; Plaxco, K. W., A Biomimetic Phosphatidylcholine-Terminated Monolayer Greatly Improves the In Vivo Performance of Electrochemical Aptamer-Based Sensors. *Angewandte Chemie International Edition* **2017,** *56* (26), 7492-7495.
18. Li, H.; Dauphin-Ducharme, P.; Ortega, G.; Plaxco, K. W., Calibration-Free Electrochemical Biosensors Supporting Accurate Molecular Measurements Directly in Undiluted Whole Blood. *Journal of the American Chemical Society* **2017,** *139* (32), 11207-11213.
19. Asai, K.; Yamamoto, T.; Nagashima, S.; Ogata, G.; Hibino, H.; Einaga, Y., An electrochemical aptamer-based sensor prepared by utilizing the strong interaction between a DNA aptamer and diamond. *Analyst* **2020,** *145* (2), 544-549.
20. Li, S.; Lin, L.; Chang, X.; Si, Z.; Plaxco, K. W.; Khine, M.; Li, H.; Xia, F., A wrinkled structure of gold film greatly improves the signaling of electrochemical aptamer-based biosensors. *RSC Advances* **2021,** *11* (2), 671-677.
